# Targeted Intra-Articular PRGF Delivery Using Enzyme-Powered Nanobots for Chondral Lesions: A Prospective Experimental Study with Biomarker Evaluation in Rabbits (*Oryctolagus cuniculus*)

**DOI:** 10.3390/ani16132010

**Published:** 2026-07-01

**Authors:** Emma Martins, Belén Cuervo, María Gemma Velasco-Martínez, Rocío Colomer-Selva, María Descalzo-Navarro, Beatriz Mena-Moros, Elena Damiá, Ángel María Hernández-Guerra, José-María Carrillo, Mónica Rubio, Joaquín Sopena

**Affiliations:** 1 Biomed Surgery Group (Bioregenerative Medicine and Applied Surgery), Group School Veterinary Medicine, Universidad Cardenal Herrera-CEU, CEU Universities, C/Tirant lo Blanc, 7, Alfara del Patriarca, 46115 Valencia, Spain; emma.martins1@uchceu.es (E.M.); gemma.velascomartinez@uchceu.es (M.G.V.-M.); rocio.colomer@uchceu.es (R.C.-S.); maria.descalzonavarr1@uchceu.es (M.D.-N.); beatriz.menamoros@uchceu.es (B.M.-M.); elena.damia@uchceu.es (E.D.); angelhdez@uchceu.es (Á.M.H.-G.); jcarrill@uchceu.es (J.-M.C.); mrubio@uchceu.es (M.R.); jsopena@uchceu.es (J.S.); 2 García Cugat Foundation CEU-UCH Chair of Medicine and Regenerative Surgery, Universidad Cardenal Herrera-CEU, CEU Universities, 46115 Valencia, Spain

**Keywords:** nanobots, urease, PRGF, osteoarthritis, chondral defect, hyaluronic acid, C2C, FRAP, oxidative stress, rabbit model

## Abstract

Osteoarthritis (OA) is the most prevalent degenerative joint disease in both humans and companion animals, causing progressive cartilage destruction for which no curative treatment currently exists. Plasma rich in growth factors (PRGF) has shown chondroprotective effects in veterinary and preclinical models, but its efficacy is limited by the viscosity of synovial fluid and rapid degradation of growth factors within the joint. This study aimed to evaluate the effect of intra-articular co-administration of PRGF and urease-powered enzymatic nanobots on serum biomarkers of inflammation, oxidative stress, and cartilage metabolism in a rabbit (*Oryctolagus cuniculus*) full-depth chondral defect model (approval code: CEEA 2023-VSC-PEA-0242). Forty-one New Zealand White rabbits were allocated to a Control group (PRGF alone; *n =* 9 evaluable) or a Nanobots group (PRGF + nanobots; *n* = 24 evaluable). Twelve serum biomarkers were measured at baseline and days 28, 56, and 84. The Nanobots group showed markedly elevated serum hyaluronic acid at all timepoints (*p* < 0.006), significantly lower type II collagen cleavage neoepitope at day 28 (*p* = 0.018), persistently lower ferric reducing ability of plasma (*p* < 0.003), and higher C-reactive protein at days 56 and 84 (*p* < 0.035). These results provide preliminary in vivo evidence that urease-powered nanobots produce a distinct biomarker profile when combined with PRGF in a rabbit chondral defect model, with potential translational relevance for veterinary orthopedic therapy.

## Simple Summary

Joint degeneration is one of the most common and painful conditions affecting dogs, cats, and humans, and there is currently no treatment that can fully repair damaged cartilage. One promising approach is to inject a preparation made from the patient’s own blood, called plasma rich in growth factors (PRGF), directly into the affected joint to promote healing. However, a major obstacle is that the thick fluid inside joints limits how far the injected substance can spread, reducing its effectiveness. In this study, we tested with nanobots, powered by a natural enzyme called urease could act as active carriers to improve the distribution of PRGF inside the joint. We surgically created a cartilage defect in both stifle joints of 41 rabbits, of whom 8 were excluded due to intercurrent health issues unrelated to the experimental treatment, resulting in 33 evaluable animals, which then received either PRGF alone or PRGF combined with nanobots. We measured several substances in their blood over three months to assess the biological response. Rabbits that received nanobots showed a different pattern of blood markers related to joint health, cartilage breakdown, and the body’s antioxidant defenses. These findings suggest that nanobots may improve the way PRGF works inside the joint, which could benefit dogs and other companion animals with joint disease in the future.

## 1. Introduction

Osteoarthritis (OA) is the most prevalent degenerative joint disease in companion animals and one of the leading causes of chronic pain and reduced quality of life in dogs and cats. In dogs, OA has been estimated to affect approximately 20% of animals aged one year or older, rising to over 80% in geriatric patients, and represents at least 80% of lameness and joint disease cases in clinical practice [1,2]. Notably, recent evidence suggests that the actual prevalence may be substantially higher, as radiographic signs of OA have been identified in 39.8% of young dogs aged 8 months to 4 years [3]. In cats, OA prevalence ranges from 16% to 91% depending on the population studied, with the highest rates reported in older individuals [4,5]. Despite its high prevalence, OA in companion animals remains frequently underdiagnosed and undertreated, representing a major unmet clinical need in small animal veterinary medicine. OA is characterized by progressive degeneration of articular cartilage, synovial inflammation, and subchondral bone remodeling, leading to pain, stiffness, and functional impairment, pathophysiological features shared across species [6,7,8]. OA also represents a major global health burden in humans, affecting over 530 million people worldwide and projected to increase significantly in the coming decades due to ageing populations and rising obesity rates [6,7]. Given the shared pathophysiological features of OA across species and the absence of effective disease-modifying treatments in both veterinary and human medicine, findings from this study may also contribute to the broader translational understanding of novel intraarticular delivery strategies for joint disease.

On a cellular level, OA progression involves a phenotypic switch of articular chondrocytes toward hypertrophy, resembling endochondral ossification and resulting in cartilage matrix breakdown [9,10]. Oxidative stress and the accumulation of reactive oxygen species (ROS) promote chondrocyte senescence, apoptosis, and matrix degradation, contributing to irreversible cartilage deterioration [11,12]. This disruption of redox homeostasis—reflected by changes in Ferric Reducing Ability of Plasma (FRAP), Trolox Equivalent Antioxidant Capacity (TEAC), Advanced Oxidation Protein Product (AOPP), Paraoxonase-1 (PON1) and thiols—is increasingly recognized as a major driver of OA progression [13,14].

Current management of OA involves a multimodal approach focused on symptom relief, inflammation control, and cartilage preservation, including Non-Steroidal Anti-Inflammatory Drugs (NSAIDs), Symptomatic Slow-Acting Drugs for Osteoarthritis (SYSADOA), intraarticular glucocorticoid and hyaluronic acid (HA) injections, and physiotherapy [15]. Regenerative interventions including Platelet-Rich Plasma (PRP), Bone Marrow Aspirate Concentrate (BMAC), and Mesenchymal Stem Cells (MSCs) have shown promising clinical outcomes in veterinary orthopedic practice, with intraarticular PRP demonstrating significant improvements in pain and function in dogs with stifle and hip OA [16,17,18,19,20]. Despite these advances, no treatment can fully restore damaged cartilage or achieve true osteochondral regeneration [21].

Plasma rich in growth factors (PRGF), an autologous preparation concentrating Transforming Growth Factor Beta (TGF-β), Insulin-like Growth Factor-1 (IGF-1), Platelet-Derived Growth Factor (PDGF) and Vascular Endothelial Growth Factor (VEGF), has demonstrated chondroprotective and regenerative effects in preclinical and clinical settings [22,23,24]. Torres-Torrillas et al. (2021) [24], from the same research group, demonstrated that combined Intra-articular (IA) + Intraosseous (IO) PRGF infiltration in a rabbit stifle joint chondral defect model produced significantly lower Type II Collagen Cleavage Neoepitope (C2C) at day 84 and transiently elevated HA at day 56, suggesting enhanced cartilage repair. However, a critical limitation of all IA-delivered biologics is their restricted penetration into deeper cartilage layers due to the viscosity of synovial fluid (SF) and rapid enzymatic degradation of growth factors in the joint environment [25,26].

Given the complexity of OA pathophysiology and the need to monitor disease progression and treatment response objectively, serum biomarkers represent an essential tool in experimental and clinical research. Biomarkers are defined as objectively measurable characteristics that indicate normal biological processes, pathological processes, or pharmacological responses to an intervention [27]. In OA, biomarkers are classified using the framework Burden of disease, Investigative, Prognostic, Efficacy of intervention, Diagnostic (BIPED), which guides their application across research and clinical contexts [27,28]. The most clinically validated serum biomarkers in OA include: (1) cartilage degradation markers such as C2C, which reflects Matrix Metalloproteinase Protein (MMP) mediated breakdown of articular cartilage and is upregulated in early OA [29,30]; (2) synovial markers such as HA, whose serum concentration rises in OA in proportion to synovitis severity and disease progression [31,32]; (3) inflammatory markers such as C-reactive protein (CRP) and haptoglobin (Hp), acute-phase reactants that rise in response to joint inflammation and are used to monitor systemic inflammatory status [33]; and (4) redox-state markers including FRAP, TEAC, AOPP, PON1 and total thiols, which collectively characterize the balance between oxidative damage and antioxidant defense—a balance shown to be disrupted in OA [12,13,14]. The simultaneous measurement of biomarkers from all four categories provides a comprehensive biochemical portrait of the joint response to both injury and treatment.

Nanorobotics offers transformative potential in biomedical science [34,35]. Enzyme-powered nanomotors (NM) are particularly relevant for intraarticular applications: passive nanoparticles struggle to penetrate viscous SF, rich in HA and glycoproteins, thereby restricting diffusion and local drug retention [36]. Ruiz-González et al. (2024) [21] demonstrated that coordinated swarms of urease- and hyaluronidase-based nanomotors (HyaNM) can synergistically reduce SF viscosity and increase macromolecular diffusion, providing a major step toward efficient intraarticular delivery. The European Research council (ERC)-funded OrthoBots project (CORDIS, 2024) has specifically identified urease-powered nanobots as a promising platform for enhancing growth factor transport in the synovial joint environment.

The present study constitutes the first in vivo experimental trial combining urease-powered enzymatic nanobots with PRGF in a rabbit chondral defect model, measuring 12 serum biomarkers across four timepoints over 84 days. The ultimate goal of this research is to develop an improved intraarticular delivery strategy for PRGF that could be translated into clinical veterinary practice for the treatment of OA in companion animals.

## 2. Materials and Methods

### 2.1. Ethical Approval and Study Design

This study was approved by the Ethics Committee of Animal Welfare (CEEA) of CEU Cardenal Herrera University (approval code: 2023-VSC-PEA-0242), in compliance with Spanish Policy for Animal Protection (Real Decreto 53/2013, modified by Real Decreto 1386/2018) transposing European Union Directive 2010/63/EU. This study was conducted and reported in accordance with the ARRIVE 2.0 guidelines (Animal Research: Reporting of In Vivo Experiments; https://doi.org/10.1371/journal.pbio.3000410). A prospective, controlled experimental study was designed to evaluate the effect of PRGF combined with urease-powered nanobots on chondral lesion repair in a rabbit model, assessed through serum biomarkers.

### 2.2. Animals

A total of 41 healthy female New Zealand White rabbits (*Oryctolagus cuniculus*), 6 months of age, mean body weight 3.57 ± 0.33 kg, were obtained from the Universidad Politécnica de Valencia and specifically bred for experimental research. Animals were individually housed with free access to water and food *ad libitum* under controlled environmental conditions (18–22 °C). An acclimatization period of one week was established prior to the experimental procedures. Animals were monitored daily for pain (Rabbit Grimace Scale), infection, and weight changes throughout the study. Animals were excluded from the study if they presented any clinical abnormality detected during the pre-surgical examination, including signs of infection, musculoskeletal pathology, or abnormal baseline laboratory values. The New Zealand White rabbit was selected as the experimental model for this study based on its well-established use in chondral repair research, the accessibility and size of its stifle joint for surgical intervention, and the availability of validated serum biomarker assays in this species. The rabbit chondral defect model is widely accepted as a relevant preclinical model for the evaluation of cartilage repair strategies, with documented translational applicability to companion animal orthopedic medicine.

### 2.3. Study Groups

The experimental design was based on a direct comparison between PRGF alone and PRGF combined with urease-powered nanobots, as the primary aim of the study was to evaluate whether nanobots could enhance or potentiate the biological effects of PRGF within the joint. It is important to note that nanobots do not constitute a therapeutic agent per se; rather, they act as active carriers designed to improve the distribution and penetration of PRGF throughout the intra-articular space and into the target tissue, including the subchondral bone. All animals with a surgically induced defect therefore received PRGF as a minimum standard of care. Animals were randomly allocated using simple randomization (Microsoft Excel random number function) into two groups:

- Control group (CT): received a single intraarticular (IA) infiltration of PRGF only in both stifle joints.
- Nanobots group (NB): received a single IA infiltration of PRGF combined with urease-powered enzymatic nanobots (suspended in Phosphate-Buffered Saline (PBS) + urea solution), in both stifle joints.

**Figure 1:**
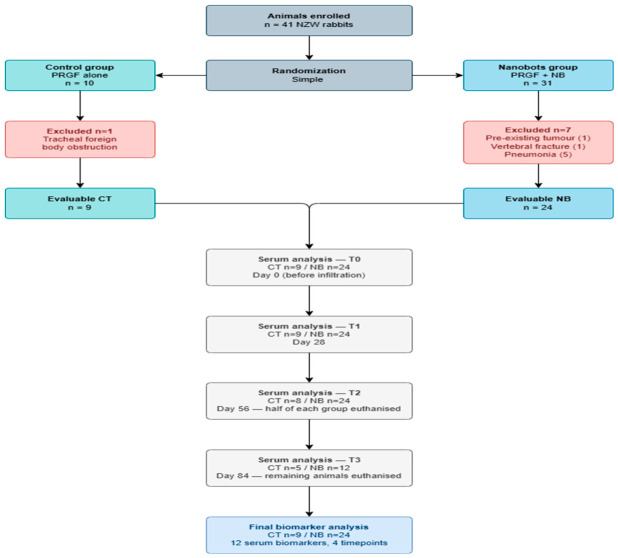
Study flow diagram showing animal enrolment, group allocation, exclusions, and number of animals available at each timepoint for serum biomarker analysis.

Of the 41 animals enrolled, 8 were excluded from analysis due to death unrelated to the experimental treatment: in the Nanobots group (*n* = 31 enrolled), one animal was excluded on the day of surgery due to the incidental finding of a pre-existing stifle joint tumor, one was sacrificed on day 5 from vertebral fracture, and five died from pneumonia. Pneumonia was confirmed by necropsy in all five cases. Deaths occurred between days 10 and 14 post-surgery, after the animals had received experimental treatment (*n* = 7 total exclusions). In the Control group (*n* = 10 enrolled), one animal died due to tracheal foreign body obstruction (*n* = 1 exclusion). None of the deaths were considered treatment related. The final evaluable sample for serum biomarker analysis comprised 9 Control and 24 Nanobots animals (Figure 1).

### 2.4. PRGF Preparation

PRGF-Endoret^®^ technology (BTI Biotechnology Institute, Vitoria, Spain) was used to obtain autologous PRGF. Under premedication with a balanced anesthesia protocol: dexmedetomidine (0.05 mg/kg Intramuscular (IM); Dexdomitor^®^, Zoetis, Parsippany, NJ, USA) with ketamine (10 mg/kg IM; Ketamidor^®^, Ecuphar/ETViva, Barcelona, Spain) and with morphine (1 mg/kg IM; Morfina B. Braun^®^ 20 mg/mL, B. Braun, Melsungen, Germany), 10 mL of blood was collected from the auricular artery with 23G catheter (Venofix^®^ 23G, B. Braun, Melsungen, Germany) in sodium citrate 3.8% vacutainer tubes, centrifuged at 460× *g* for 8 min (PRGF^®^ System III, BTI, Vitoria, Spain). The PRGF fraction was collected and activated with 10% CaCl_2_ (50 µL/mL) immediately before infiltration.

### 2.5. Nanobots

Urease-powered enzymatic nanobots were developed and provided by the Smart Nano-bio-devices Group (IBEC, Barcelona, Spain). These nanoscale devices (1–100 nm) are propelled by surface-bound urease, catalyzing the hydrolysis of urea (present in PBS + urea solution) into CO_2_ and NH_3_, generating asymmetric bubble-driven propulsion. Nanobots were combined with PRGF immediately prior to infiltration.

### 2.6. Surgical Procedure

**Figure 2:**
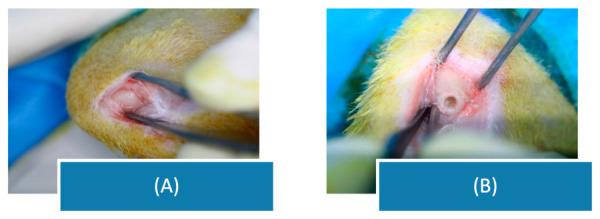
Macroscopic appearance of the medial femoral condyle of the rabbit stifle joint: (**A**) prior to surgical creation of the chondral defect, showing intact articular cartilage; (**B**) following creation of the full-thickness chondral defect, exposing the subchondral bone.

Animals were premedicated IM with dexmedetomidine (0.05 mg/kg IM; Dexdomitor^®^, Zoetis, Parsippany, NJ, USA) with ketamine (10 mg/kg IM; Ketamidor^®^, Ecuphar/ETViva, Barcelona, Spain) and with morphine (1 mg/kg IM; Morfina B. Braun^®^ 20 mg/mL, B. Braun, Melsungen, Germany). An IV catheter 20G (Vasofix^®^ Safety 20G, B. Braun, Melsungen, Germany) was placed in the auricular vein for administration of propofol (5 mg/kg IV; Propofol-Lipuro^®^ 10 mg/mL, B. Braun, Melsungen, Germany) and fentanyl as needed (0.025 mg/kg IV; Fentanest^®^, Janssen, Madrid, Spain), during the induction phase. Anesthesia maintenance was achieved with isoflurane (2%). Throughout all procedures, the following anesthesia parameters were continuously monitored: heart rate, respiratory rate, arterial oxygen saturation (SpO_2_), end-tidal CO_2_ (EtCO_2_), body temperature, and blood pressures. The medial aspect of both stifle joints was clipped and prepared aseptically. All surgical procedures and infiltrations were performed by the same experienced surgeon to minimize operator variability. A 10 mm incision was made at the margin of the medial femoral condyle (No. 11 scalpel blade), followed by arthrotomy. A full-thickness chondral defect extending to the subchondral bone of 5 mm in diameter was created with a calibrated drill guide (Figure 2). Wound closure was performed in layers (3/0 polyglyconate; Novosyn^®^ Quick, B-Braun, Melsungen, Germany). The procedure was repeated on the contralateral stifle joint. Immediately after closure, IA infiltration was performed (0.25 mL per stifle joint) with a 23G needle (Sterican^®^ 23G, B. Braun, Melsungen, Germany) inserted laterally into the patellar tendon. In the Control group, each stifle joint received a single intraarticular infiltration of 0.25 mL of PRGF. In the Nanobots group, each stifle joint received a combined intra-articular infiltration of 0.25 mL of PRGF mixed with 0.2 mL of urease-powered nanobots suspended in PBS + urea solution, for a total infiltration volume of 0.45 mL per joint. The two components were combined immediately prior to infiltration.

### 2.7. Postoperative Care

All animals received meloxicam (0.3 mg/kg SC q24h; Metacam^®^, Boehringer Ingelheim, Germany) and enrofloxacin (10 mg/kg SC q24h; Baytril^®^, Elanco, Indianapolis, IN, USA) for a minimum of 7 days. Rescue analgesia (buprenorphine 0.01 mg/kg SC q8h) was given when Rabbit Grimace Scale scores were ≥4, but that was not required in any animal. No clinical signs of systemic toxicity, adverse inflammatory reactions, or treatment-related complications were observed in any animal throughout the 84-day study period. All animal deaths recorded during the study were unrelated to the experimental treatment, as described in the Study Groups section.

### 2.8. Euthanasia and Sample Collection

Half of each group was euthanized on day 56 and the remainder on day 84. Animals received premedication with dexmedetomidine (0.05 mg/kg IM; Dexdomitor^®^, Zoetis, Parsippany, NJ, USA) with ketamine (10 mg/kg IM; Ketamidor^®^, Ecuphar/ETViva, Barcelona, Spain) and euthanized by intracardiac pentobarbital sodium (150 mg/kg; Dolethal^®^, Vétoquinol, Lure, France). Both stifle joints (right and left) were collected by femoral and tibial osteotomy following euthanasia and fixed in 10% buffered formalin for preservation.

Blood samples (3.5 mL) were collected from the auricular artery with a 23G vacuntainer catheter (Venofix^®^ 23G, B. Braun, Melsungen, Germany) at T0 (day of surgery, before infiltration), T1 (day 28), T2 (day 56), and T3 (day 84). Serum was obtained by centrifugation (3000× *g*, 5 min) and stored at −80 °C until analysis.

### 2.9. Serum Biomarker Analysis

All serum biomarker analyses were performed at Interdisciplinary Laboratory of Clinical Analysis (Interlab-UMU) at the University of Murcia for processing by personnel blinded to group allocation. Frozen serum samples were shipped on dry ice and processed according to standardized protocols. Twelve serum biomarkers were measured, grouped into three functional categories:

- Inflammatory markers: C-reactive protein (CRP, µg/mL), haptoglobin (Hp, mg/dL), total protein (PROT, g/dL), albumin (ALBU, g/dL), and globulins (GLOB, g/dL). These were determined by automated immunochemical and colorimetric methods using an Olympus AU400 chemistry Analyzer (Beckman Coulter, Brea, CA, USA) with validated commercial reagents (University of Murcia, Prof. J.J. Cerón).
- Redox-state markers: paraoxonase-1 (PON1, International Units IU/mL), total thiols (Thiol, µmol/L), ferric reducing ability of plasma (FRAP, mmol/L), Trolox equivalent antioxidant capacity (TEAC, mmol/L), and advanced oxidation protein products (AOPP, µmol/L). These were determined by spectrophotometric colorimetric and enzymatic methods using a BioTek microplate reader (BioTek Instruments, Winooski, VT, USA) at the Interdisciplinary Laboratory of Clinical Analysis, University of Murcia (Prof. J.J. Cerón).
- Cartilage-specific biomarkers: type II collagen cleavage neoepitope (C2C, ng/mL) by sandwich Enzime-Linked immunosorbent assay (ELISA) (MyBioSource, San Diego, CA, USA); serum hyaluronic acid (HA, ng/mL) by competitive ELISA (TECO Medical, TE1017-2, Sisseln, Switzerland). Both ELISAS were performed at the University of Murcia laboratory of Prof. J.J. Cerón.

### 2.10. Statistical Analysis

Data were analyzed using SPSS v.29.0.20 (20). Normality was assessed with the Shapiro–Wilk test and homogeneity of variances was evaluated using Levene’s test. All twelve serum biomarkers showed non-normal distributions at one or more timepoints, confirming non-normal distributions across the dataset. Between-group comparisons at individual timepoints were performed using the Mann–Whitney U test for two-group comparisons and the Kruskal–Wallis test for multiple comparisons, given the non-normal distribution of data as assessed by the Shapiro–Wilk test. To evaluate the overall longitudinal trajectory of each biomarker and assess whether the pattern of change over time differed between groups, a repeated measures ANOVA model was additionally applied, including group (Control vs. Nanobots), time (T0, T1, T2, T3), and the group × time interaction as factors, thereby accounting for within-subject correlation across repeated measurements. Statistical significance: *p* ≤ 0.05, following the conventional threshold in biomedical and veterinary experimental research. Data expressed as median (IQR).

## 3. Results

The final evaluable sample consisted of 9 Control rabbits (CT; PRGF alone) and 24 Nanobots rabbits (NB; PRGF + urease nanobots). Sample sizes per timepoint were: T0 CT = 9/NB = 24; T1 CT = 9/NB = 24; T2 CT = 8/NB = 24; T3 CT = 5/NB = 12, reflecting the planned euthanasia schedule. All between-group comparisons were performed with the Wilcoxon rank-sum test after normality assessment by Shapiro–Wilk and homogeneity of variances assessment by Levene’s test. All twelve serum biomarkers failed the Shapiro–Wilk normality test at one or more timepoints, confirming non-normal distributions across the dataset and justifying the use of non-parametric statistical tests throughout the analysis. Complete descriptive statistics and *p*-values are presented in Table 1.

### 3.1. Serum Hyaluronic Acid (HA)

**Figure 3:**
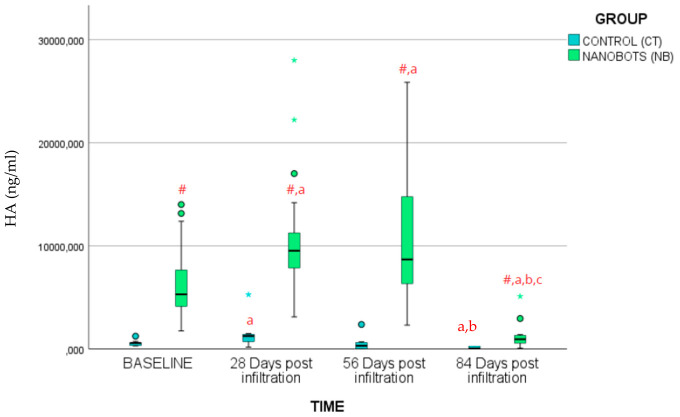
Serum hyaluronic acid (HA ng/mL) before and after infiltration. Box= IQR; line = median; * = extrema outliers; • = medium outliers; # = significant difference between group (*p*-value < 0.05); a = significant difference with T0 (*p*-value < 0.05), b = significant difference with T1 (*p*-value < 0.05); c = significant difference with T2 (*p*-value < 0.05), within the same group.

Serum HA concentrations showed a marked and consistent elevation in the NB group compared to the CT group at all timepoints (Figure 3). At baseline (T0), prior to surgical intervention and infiltration, median HA was already higher in the NB group (5255.08 ng/mL [IQR 4131.26–6951.63]; *n* = 24) than in CT (572 ng/mL [IQR 329.7–572.19]; *n* = 9; *p* < 0.001). In the NB group, HA concentrations rose progressively from T0 to T2, reaching a peak at day 56 (308.85 ng/mL [IQR 33.75–583.46]), before declining markedly at T3 (39.85 ng/mL [IQR 9.05–316.45]; *n* = 12). By contrast, CT group values remained low and relatively stable throughout the study (T1: 1244.45 ng/mL [IQR 708.85–1413.25]; T2: 308.85 ng/mL [IQR 33.75–583.46]; T3: 39.85 ng/mL [IQR 9.05–316.45]). Significant between-group differences were detected at all four timepoints: T0 (*p* < 0.001), T1 (*p* < 0.001), T2 (*p* < 0.001), and T3 (*p* = 0.006) (Figure 3).

### 3.2. Type II Collagen Cleavage Neoepitope (C2C)

**Figure 4:**
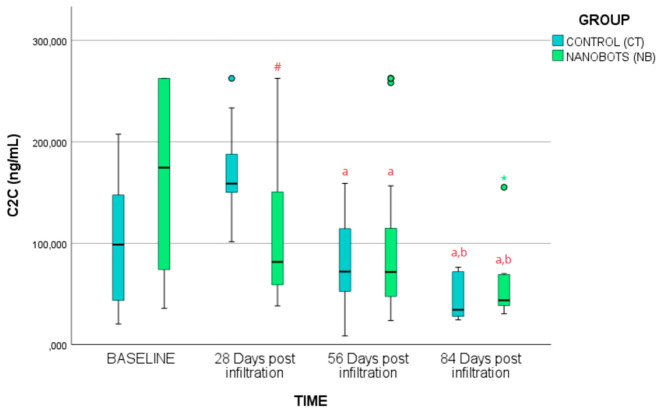
Serum type II collagen cleavage Neoepitope (C2C ng/mL) before and after infiltration. Box = IQR; line = median; * = extrema outliers; • = medium outliers; # = significant difference between group (*p*-value < 0.05); a = significant difference with T0 (*p*-value < 0.05), b = significant difference with T1 (*p*-value < 0.05) within the same group.

Serum C2C concentrations showed an initial rise in both groups from baseline to day 28, followed by a progressive decline through day 84 (Figure 4). At baseline (T0), median C2C was numerically higher in the NB group (174.57 ng/mL [IQR 75.47–262.5]; *n* = 24) than in CT (98.77 ng/mL [IQR 43.36–147.52]; *n* = 9), although this difference did not reach statistical significance (*p* = 0.052). At T1 (day 28), both groups showed elevated C2C relative to baseline; however, the NB group demonstrated significantly lower values than CT (81.73 ng/mL [IQR 59.08–140.31] vs. 158.87 ng/mL [IQR 150.14–187.7]; *p* = 0.018). From T1 onwards, C2C declined progressively in both groups. At T2 (day 56), no significant between-group difference was detected (71.77 ng/mL [IQR 47.41–110.6] vs. 72.15 ng/mL [IQR 59.16–98.95]; *p* = 1.000), nor at T3 (day 84) (43.52 ng/mL [IQR 39.0–68.28] vs. 34.1 ng/mL [IQR 27.64–72.07]; *p* = 0.442) (Figure 4).

### 3.3. C-Reactive Protein (CRP)

**Figure 5:**
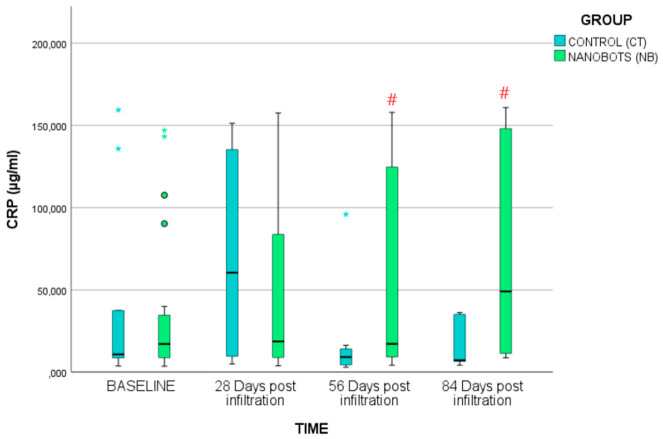
Serum C-reactive protein (CRP µg/mL) before and after infiltration. Box = IQR; line = median; * = extrema outliers; • = medium outliers; # = significant difference between group (*p*-value < 0.05).

Serum CRP concentrations rose in both groups at T1 (day 28) relative to baseline, consistent with the acute inflammatory response to surgical trauma. At T0, no significant between-group difference was observed (10.70 µg/mL [IQR 8.5–37.3] vs. 17 µg/mL [IQR 9.12–34.45]; CT vs. NB; *p* = 0.793), nor at T1 (60.5 µg/mL [IQR 9.5–135.2] vs. 18.55 µg/mL [IQR 8.95–78.60]; *p* = 0.613). From T1 onwards, the two groups diverged markedly: CT showed a progressive decline through T2 and T3, while NB maintained elevated values. At T2 (day 56), CRP was significantly higher in the NB group (17.10 µg/mL [IQR 9.57–123.4] vs. 9.10 µg/mL [IQR 4.47–12.90]; *p* = 0.035), and this difference was further accentuated at T3 (49.10 µg/mL [IQR 12.28–146.22] vs. 7.10 µg/mL [IQR 6.30–34.90]; *p* = 0.019) (Figure 5).

### 3.4. Haptoglobin (Hp)

**Figure 6:**
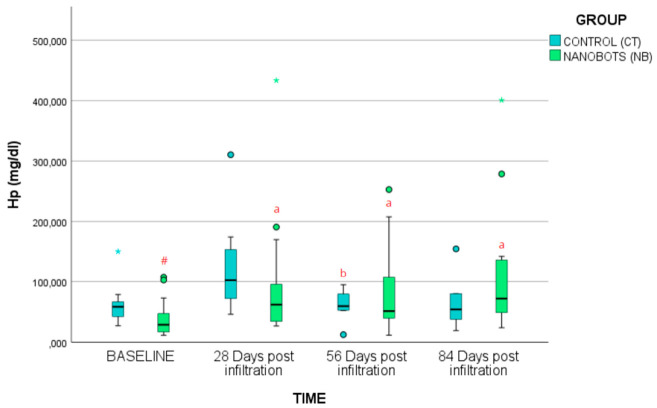
Serum haptoglobin (Hp mg/mL) before and after infiltration. Box = IQR; line = median; * = extrema outliers; • = medium outliers; # = significant difference between group (*p*-value < 0.05); a = significant difference with T0 (*p*-value < 0.05), b = significant difference with T1 (*p*-value < 0.05) within the same group.

Serum Hp concentrations rose in both groups at T1 (day 28) relative to baseline, consistent with the acute-phase response to surgical trauma, before declining toward baseline levels at T2 and T3. At T0, Hp was significantly lower in the NB group (28.75 mg/dL [IQR 16.82–46.54]; *n* = 24) than in CT (58.35 mg/dL [IQR 42.34–66.41]; *n* = 9; *p* = 0.018). At T1, both groups showed elevated Hp relative to baseline; a non-significant trend toward lower values was observed in NB (61.98 mg/dL [IQR 34.44–92.99] vs. 102.22 mg/dL [IQR 71.86–153.34]; *p* = 0.055). No significant between-group differences were detected at T2 (51.19 mg/dL [IQR 39.92–102.30] vs. 59.49 mg/dL [IQR 52.97–76.42]; *p* = 0.949) or T3 (71.99 mg/dL [IQR 51.02–113.29] vs. 53.98 mg/dL [IQR 37.75–80.06]; *p* = 0.442) (Figure 6).

### 3.5. Ferric Reducing Ability of Plasma (FRAP)

FRAP was significantly lower in the NB group at all four timepoints: T0 (0.29 mmol/L [IQR 0.27–0.31] vs. 0.35 mmol/L [IQR 0.34–0.35]; *p* < 0.001), T1 (0.34 mmol/L [IQR 0.33–0.35] vs. 0.30 mmol/L [IQR 0.26–0.31]; *p* = 0.001), T2 (0.35 mmol/L [IQR 0.31–0.37] vs. 0.42 mmol/L [IQR 0.39–0.43]; *p* = 0.003), and T3 (0.26 mmol/L [IQR 0.25–0.28] vs. 0.36 mmol/L [IQR 0.32–0.37]; *p* = 0.001).

### 3.6. TEAC (Trolox Equivalent Antioxidant Capacity)

TEAC was significantly lower in the NB group at T0 (0.67 mmol/L [IQR 0.65–0.70] vs. 0.74 mmol/L [IQR 0.73–0.74]; *p* < 0.001). No significant differences were detected at T1 (0.76 mmol/L [IQR 0.72–0.78] vs. 0.73 mmol/L [IQR 0.70–0.74]; *p* = 0.072), T2 (0.78 mmol/L [IQR 0.72–0.81] vs. 0.80 mmol/L [IQR 0.79–0.81]; *p* = 0.480), or T3 (0.73 mmol/L [IQR 0.69–0.75] vs. 0.76 mmol/L [IQR 0.74–0.77]; *p* = 0.234).

### 3.7. Advanced Oxidation Protein Products (AOPP)

AOPP was significantly higher in the NB group at T0 (56.15 µmol/L [IQR 47.78–68.53] vs. 41.00 µmol/L [IQR 38.90–45.80]; *p* < 0.001). No significant differences were detected at T1 (48.85 µmol/L [IQR 43.95–55.65] vs. 47.70 µmol/L [IQR 45.00–49.70]; *p* = 0.686), T2 (48.45 µmol/L [IQR 42.18–59.38] vs. 57.00 µmol/L [IQR 48.02–64.53]; *p* = 0.184), or T3 (53.70 µmol/L [IQR 45.60–62.52] vs. 48.40 µmol/L [IQR 38.00–49.50]; *p* = 0.104).

### 3.8. Paraoxonase-1 (PON1)

**Figure 7:**
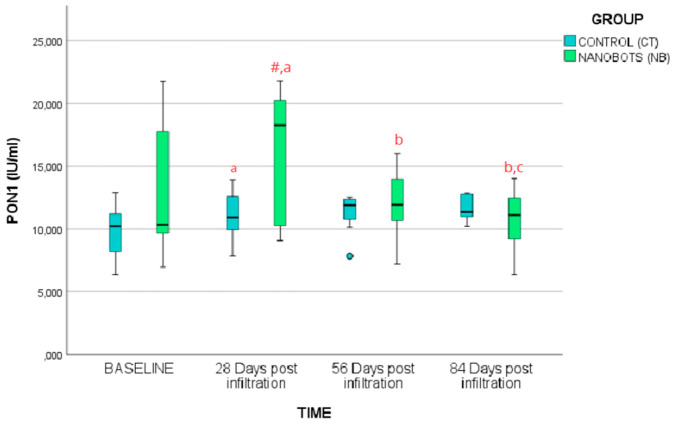
Serum paraoxonase-1 (PON1 IU /mL) before and after infiltration. Box = Interquartile Range (IQR); line = median; • = medium outliers; # = significant difference between group (*p*-value <0.05); a = significant difference with T0 (*p*-value < 0.05), b = significant difference with T1 (*p*-value < 0.05); c = significant difference with T2 (*p*-value < 0.05), within the same group.

Serum PON1 concentrations were comparable between groups at baseline (10.36 IU/mL [IQR 9.70–17.65] vs. 10.24 IU/mL [IQR 8.24–11.21]; NB vs. CT; *p* = 0.322). At T1 (day 28), PON1 was significantly higher in the NB group (18.27 IU/mL [IQR 10.29–20.13] vs. 10.93 IU/mL [IQR 9.93–12.58]; *p* = 0.037). From T1 onwards, values in both groups converged and no significant differences were detected at T2 (11.94 IU/mL [IQR 10.90–13.95] vs. 11.90 IU/mL [IQR 11.08–12.34]; *p* = 0.542) or T3 (11.11 IU/mL [IQR 9.26–12.49] vs. 11.35 IU/mL [IQR 10.99–12.78]; *p* = 0.460) (Figure 7).

### 3.9. Non-Significant Biomarkers: Total Protein, Albumin, Globulins, and Thiols

Total protein (PROT), albumin, globulins, and total thiols (Thiol) showed not statistically significant between-group differences at any timepoint (all *p* > 0.05; Table 1). Both groups showed expected post-surgical increases in PROT and GLOB at T1 relative to T0, followed by progressive normalization toward T3, consistent with the normal acute-phase response to surgical trauma.

## 4. Discussion

This study constitutes, to our knowledge, the first in vivo experimental evaluation of urease-powered enzymatic nanobots as facilitators of intraarticular PRGF delivery in a rabbit full-depth chondral defect model. The biomarker profile obtained reveals several biologically relevant findings with potential implications for the development of novel regenerative therapies for OA in companion animals, particularly dogs, where PRGF-based ortho-biologics are already used in clinical practice and considering the mechanistic rationale proposed for nanobot-mediated delivery. It should be noted that all biomarkers were measured in serum rather than synovial fluid. This approach was chosen due to the limited volume of synovial fluid available in rabbit joints, which makes repeated sampling technically challenging and is not well standardized in this species. While serum biomarkers cannot be assumed to reflect exclusively local intra-articular events, they have been widely used in previous studies to monitor joint-related biological changes and are considered a valid approach in this experimental context [32].

### 4.1. Serum Hyaluronic Acid: A Markedly Amplified Response

Hyaluronic acid is the most abundant glycosaminoglycan in articular cartilage and synovial fluid, with central roles in joint lubrication, viscoelasticity, and chondrocyte homeostasis [37]. Serum HA, produced predominantly by synoviocytes, reflects the extent of synovial membrane activity and has been validated as a marker of OA severity and treatment response in humans, dogs, and rabbits [31,32]. In OA, synovial fluid HA decreases in molecular weight and concentration while serum HA rises in proportion to synovitis [38].

Following full-depth chondral injury, serum HA is expected to rise because of increased synoviocyte activity in response to joint damage, and to subsequently decline as the repair process resolves and synovial homeostasis is restored [32,38]. The present results are consistent with this expected trajectory in the NB group, which showed a progressive rise from baseline to day 56 followed by a marked decline on day 84, while the CT group remained at low stable values throughout the study. The HA results of the present study are different from those reported by Torres-Torrillas et al. (2021) [24], who used the same rabbit chondral defect model with combined IA + IO PRGF and observed a transient, significant HA elevation only at day 56 in the treatment group (3119 vs. 974 ng/mL; *p* = 0.021), interpreted as a marker of accelerated chondrogenesis, followed by normalization at day 84. In contrast, the NB group in the present study showed HA values of 6243 ng/mL already at T0, rising to over 10,000 ng/mL at T1 and T2, values approximately three times higher than those reported by Torres-Torrillas et al. [24] at their peak. The significant T0 difference, occurring before surgery and infiltration, is most likely attributable to the unequal group allocation (*n* = 9 vs. *n* = 24) causing a chance baseline imbalance, as discussed in the Limitations section. However, the progressive rise from T0 to T2 and the subsequent decline at T3 describes a biphasic pattern that is consistent with an accelerated and amplified chondrogenic synovial response. Ruiz-González et al. (2024) [21] demonstrated that urease NM (UrNM) swarms actively alter the biophysical properties of SF and enhance macromolecular diffusion; this mechanism could facilitate broader synoviocyte stimulation by PRGF growth factors compared to PRGF alone. Importantly, Battistoni et al. (2025) [39] demonstrated in an in vitro OA inflammatory model that hyaluronic acid inclusion in collagen-based scaffolds significantly reduces MMP-13 expression at both gene and protein level, suggesting that the elevated systemic HA observed in the NB group may also reflect a chondroprotective, MMP-13-suppressing environment consistent with the C2C reduction observed simultaneously at T1. The T3 decline (from 10,684 to 1291 ng/mL) is consistent with normalization following a completed anabolic phase, as proposed by Filková et al. (2009) [38] and Vilar et al. (2016) [32], and would align with the expected trajectory of successful chondral repair.

### 4.2. C2C: Earlier Chondroprotection

C2C is a neoepitope generated by MMP-1, MMP-8, and MMP-13 mediated cleavage of type II collagen, and is a validated serum marker of active cartilage matrix degradation across multiple species including rabbits [24,29,30]. In OA, C2C is elevated in early stages and declines as the degradative substrate is progressively depleted [40].

Following surgical creation of a full-depth chondral defect, serum C2C is expected to rise acutely because of MMP-mediated type II collagen degradation triggered by the injury, and to subsequently decline progressively as the acute degradative phase resolves and repair mechanisms are established [29,40]. Consistent with this expected trajectory, both groups showed an initial rise in C2C from baseline to day 28, followed by a progressive decline through day 84. Notably, the NB group demonstrated a faster and more pronounced reduction from day 28 onwards compared to the CT group, with a significant between-group difference already apparent at T1 (*p* = 0.018), suggesting an earlier onset of chondroprotection in nanobot-treated animals. Torres-Torrillas et al. (2021) [24] reported a significant C2C reduction in the PRGF treatment group compared to control, but only on day 84 (7.26 vs. 24.15 ng/mL; *p* = 0.038). In the present study, a significant C2C reduction in the NB group was already apparent on day 28 (T1: 116.4 vs. 175.1 ng/mL; *p* = 0.018), representing a 33.5% reduction in type II collagen degradation at the critical early post-surgical phase. This earlier onset of chondroprotection is the most clinically interpretable finding of the present study, as it suggests that the addition of nanobots may accelerate the therapeutic action of PRGF growth factors, particularly TGF-β and IGF-1, which are known to suppress MMP expression and promote type II collagen synthesis [22,23]. This is mechanistically coherent with the hypothesis that nanobots enhance PRGF penetration and distribution within articular cartilage. As mentioned earlier, Battistoni et al. (2025) [39] demonstrated in an inflammatory OA in vitro model that hyaluronic acid within collagen scaffolds significantly reduces MMP-13 expression, which is the principal collagenase responsible for C2C generation. The simultaneous elevation of HA and reduction of C2C at T1 in the NB group is consistent with this anti-MMP-13 mechanism operating in an HA-rich articular environment [39]. Vilar et al. (2016) [32] demonstrated in OA dogs treated with MSC + PRGF that early C2C reduction correlated inversely with functional improvement measured by force plate analysis, further supporting C2C as a sensitive early efficacy biomarker. The absence of significant C2C differences at T2 and T3 likely reflects the progressive convergence of both groups as the acute surgical injury resolves.

### 4.3. CRP: Late-Phase Elevation in the Nanobots Group

CRP is a hepatically produced acute-phase protein responding to Interleukin-6 (IL-6) and other pro-inflammatory cytokines, widely used as a systemic inflammation marker in human and veterinary OA [32,33]. The late and progressive CRP elevation in the NB group at T2 and T3 and no elevation in CT, requires careful interpretation. It may reflect a sustained low-grade systemic inflammatory response to the nanobot catalytic byproducts (CO_2_ and NH_3_), or to the immune recognition of nanobot particles themselves, a possibility that, although no clinical signs of adverse inflammation were observed in any animal, warrants investigation in future safety studies [41]. Or elevated late-phase CRP may reflect active tissue remodeling. CRP is not merely a passive marker of inflammation: growing evidence demonstrates that it undergoes context-dependent conformational changes in vivo, dissociating from its native pentameric form (pCRP) into a monomeric isoform (mCRP) at sites of tissue injury, where mCRP co-localizes with leukocytes and participates in the localized amplification and subsequent resolution of inflammation [41]. Furthermore, the 2025 review by Potempa et al. specifically documents a role for mCRP in tissue regeneration, neovascularization, and angiogenesis during wound healing [42], while cartilage repair requires a controlled pro-inflammatory environment at intermediate timepoints for matrix remodeling, angiogenic signaling, and reparative cell recruitment [33].

### 4.4. Oxidative Stress Markers: Baseline Imbalance and Safety Considerations

The three oxidative stress markers showing significant baseline differences: FRAP (lower in NB at T0, *p* < 0.001), TEAC (lower in NB at T0, *p* < 0.001), and AOPP (higher in NB at T0, *p* < 0.001). Previous studies of OA and oxidative stress have consistently reported lower PON1, FRAP, and TEAC alongside higher AOPP in OA patients compared to healthy controls [12,13,14].

The most important finding regarding oxidative stress is the absence of change: despite the lower baseline antioxidant capacity in the NB group, AOPP differences normalized after T0, and no progressive worsening of protein oxidative damage was observed over the 84-day study period. The nanobots used in this study are based on mesoporous silica nanoparticles (MSNPs), which have an established safety profile in preclinical research. Multiple in vivo studies have demonstrated that MSNPs do not alter serum albumin, total protein, or globulin concentrations at therapeutic doses, and that they are degradable and cleared within days after intravenous administration [43,44]. The Food and drugs administration (FDA) has approved colloidal silica for pharmaceutical use, and MSNPs have undergone several clinical trials with acceptable safety profiles [44]. The absence of progressive AOPP elevation in the NB group is therefore consistent with the known biocompatibility of MSNPs and supports the view that the UrNM catalytic activity did not impose a clinically significant additional oxidative burden. This suggests that the UrNM catalytic activity, generating CO_2_ and NH_3_ as byproducts [21], did not impose a clinically significant additional oxidative burden in vivo and supports the overall safety profile of the treatment. The FRAP difference persisted throughout the study, suggesting a maintained but non-worsening antioxidant gap that may reflect the constitutional baseline difference rather than an active treatment effect. In contrast, TEAC—which additionally captures lipophilic and thiol-containing antioxidants such as vitamin E, vitamin C and urate, unlike FRAP—normalized from T1 onwards, suggesting that the non-enzymatic lipophilic antioxidant fraction recovered rapidly after baseline while the broader reducing capacity measured by FRAP remained altered [11,12,14].

### 4.5. PON1: Transient Antioxidant Mobilization Day 28

PON1 is a calcium-dependent Hight-density Protein (HDL)-associated esterase/lactonase with well-established antioxidant, anti-inflammatory, and anti-atherogenic properties [45]. PON1 has been shown to regulate inflammatory signaling pathways including Nuclear factor Kappa B (NF-κB) and Mitogen-Activated Protein Kinase (MAPKs) in macrophages in vitro and in vivo [46]. Reduced PON1 activity has been reported in stifle joint OA patients in inverse correlation with radiological severity, with PON1 activity significantly lower in OA patients compared to healthy controls (*p* < 0.001), proposing a diagnostic and monitoring role for this biomarker [13,47,48].

The transient PON1 elevation in the NB group exclusively at T1 (day 28), coinciding precisely with the C2C reduction, is therefore biologically meaningful in two complementary ways. First, higher PON1 at day 28 in the NB group is consistent with a more effective suppression of early macrophage-driven inflammatory responses at the repair site, directly attenuating the pro-inflammatory cascade triggered by surgical cartilage injury [46,49]. Second, PRGF growth factors (particularly TGF-β and IGF-1) are known to modulate hepatic acute-phase protein production and could activate signaling pathways that upregulate PON1 transcription [22,50]. The return to comparable and stable PON1 levels in both groups at T2 and T3 is consistent with the resolution of the acute repair phase and the re-establishment of redox homeostasis, as PON1 activity has been shown to fluctuate transiently in response to post-surgical inflammation and to normalize as inflammatory markers decline [51].

### 4.6. Haptoglobin: Baseline Difference and Early Trend

Haptoglobin is a positive acute-phase protein in rabbits with hemoglobin-binding, anti-inflammatory, and antioxidant functions [32]. Interestingly, the trend toward lower Hp in NB at T1 (*p* = 0.055), may reflect a more rapidly resolving early inflammatory phase in nanobot-treated animals. Vilar et al. (2016) [32] reported that Hp dynamics in OA dogs correlated with functional outcomes measured by force plate analysis, which, if replicated here, would support a more favorable early inflammatory resolution profile in the NB group.

### 4.7. Non-Significant Biomarkers: Evidence Supporting Systemic Safety

The absence of significant between-group differences in total protein, albumin, globulins, and thiols at any timepoint is a relevant finding. Serum albumin and total protein are the primary conventional markers of hepatic synthetic capacity used in preclinical toxicity evaluation; a reduction in serum albumin is considered one of the criteria of severity in drug- or xenobiotic-induced liver injury, and its stability in a treatment group is widely interpreted as evidence of preserved hepatic function [52]. The stability of both markers throughout the 84-day study period in the NB group therefore indicates that the intraarticular administration of urease nanobots did not produce detectable hepatic synthetic impairment or systemic protein metabolism disturbance. Globulins, reflecting immunoglobulin and complement production, remained stable, indicating no sustained systemic immune activation attributable to the nanobot treatment. These findings are consistent with the established safety profile of MSNPs, which multiple preclinical studies have demonstrated to be safe at therapeutic doses without altering serum protein or albumin levels [43,44]. The expected post-surgical acute-phase increase in total protein and globulins at T1 in both groups confirms the adequate sensitivity of these markers and validates the biological coherence of the dataset. The absence of between-group differences in this acute-phase response further indicates that the nanobot treatment did not amplify the surgical inflammatory response at a systemic protein level.

Considered together, the biomarker profile of the NB group in the present study differs from the CT study in three key respects:

(1) a dramatically amplified and sustained serum HA response.
(2) an earlier onset of C2C reduction.
(3) a late-phase CRP elevation without parallel in prior PRGF studies.

The first two findings are biologically consistent with the hypothesis that nanobots enhance intraarticular PRGF distribution, promoting more rapid and extensive synoviocyte stimulation and earlier chondroprotection. The third finding requires further investigation to distinguish between productive remodeling and a low-grade inflammatory response to nanobots.

The present study introduces nanobots as an alternative strategy for enhanced PRGF delivery and obtains a more pronounced and earlier response than Torres-Torrillas et al. 2021 [24]. However, the pending histological data prevent definitive conclusions about treatment superiority. Future studies with histological correlation are essential to validate these preliminary findings. If confirmed, the nanobot-assisted PRGF delivery strategy described here could represent a clinically applicable and minimally invasive approach to improve the management of joint disease in dogs and other companion animals, addressing a major unmet need in veterinary orthopedic medicine.

## 5. Limitations

Several limitations of this study must be acknowledged to contextualize the results appropriately.

First, the group sizes were substantially unequal (CT *n* = 9 vs. NB *n* = 24), a consequence of the original allocation design and animal losses during follow-up. This imbalance increases the probability of random baseline differences between groups and reduces statistical power for between-group comparisons, particularly at T3 where only five Control animals were available. The significant differences observed at T0 for HA, FRAP, TEAC, AOPP, and Hp are most likely attributable to this allocation imbalance rather than to any treatment effect and should be interpreted with caution.

Second, histological evaluation of cartilage repair quality was not included in the present study. This represents an important limitation, as histological data would provide the ground truth necessary to confirm whether the biomarker changes observed—particularly the reduction in C2C and the elevation of HA in the Nanobots group—translate into actual improvements in cartilage tissue repair. The present findings should therefore be considered preliminary evidence based on systemic biomarkers, and future studies incorporating histological and macroscopic joint assessment are warranted to validate these results.

Third, as all biomarkers were measured in serum rather than synovial fluid, it cannot be excluded that some of the observed changes reflect systemic responses rather than exclusively localized intra-articular events. This should be considered when interpreting the specificity of the biomarker findings with respect to the treated joint. This approach was nonetheless chosen due to the limited volume of synovial fluid available in rabbit joints, which makes repeated sampling technically challenging and is not well standardized in this species.

Fourth, the rabbit chondral defect model represents an acute traumatic injury in clinically healthy young animals, rather than a chronic progressive chondral defect model. The biomarker findings should therefore be interpreted in the context of acute cartilage repair, and caution should be exercised when extrapolating these results to chronic degenerative joint disease settings in older patients.

Fifth, five animals in the Nanobots group died from pneumonia between days 10 and 14 post-surgery, confirmed by necropsy. Although the temporal pattern of death argues against an acute hypersensitivity reaction to the treatment, and no adverse reactions were observed in surviving animals, a causal relationship with the experimental treatment cannot be entirely excluded. Future studies should implement more rigorous peri-operative respiratory monitoring and antibiotic protocols, including culture and sensitivity testing within the rabbit population, as recommended by Crowley et al. (2021) [53].

## 6. Conclusions

This study presents the first in vivo experimental evidence for the use of urease-powered enzymatic nanobots as PRGF delivery facilitators in a rabbit chondral defect model. The principal findings are as follows:

The earlier attenuation of type II collagen degradation observed in the Nanobots group suggests that the addition of nanobots may accelerate the chondroprotective action of PRGF, which is consistent with the hypothesis that active nanobot-mediated delivery enhances the penetration and distribution of growth factors within the articular environment, including the subchondral bone. The amplified and sustained hyaluronic acid response further supports a more pronounced synoviocyte anabolic reaction in nanobot-treated animals.

The absence of systemic toxicity signals across all safety-related biomarkers supports the biocompatibility of urease-powered nanobots at the doses used, which is an essential prerequisite for their future application in veterinary orthopedic practice.

These findings should be interpreted as preliminary biomarker-based evidence, as histological confirmation of cartilage tissue outcomes remains pending. Future studies with larger and more balanced cohorts, as well as chronic chondral defect models, are needed.

## Figures and Tables

**Table 1:** Serum biomarkers values (median (IQR)) and between group T0, T1, T2 and T3.

Biomarker	CT T0	NB T0	*p*-Value T0	CT T1	NB T1	*p*-Value T1	CT T2	NB T2	*p*-Value T2	CT T3	NB T3	*p*-Value T3
**CRP (µg/mL)**	10.70 (8.50–37.30)	17.00 (9.12–34.45)	0.793	60.50 (9.50–135.20)	18.55 (8.95–78.60)	0.613	9.10 (4.47–12.90)	17.10 (9.57–123.40)	0.035 *	7.10 (6.30–34.90)	49.10 (12.28–146.22)	0.019 *
**Hp (mg/dL)**	58.35 (42.34–66.41)	28.75 (16.82–46.54)	0.018 *	102.22 (71.86–153.34)	61.98 (34.44–92.99)	0.055	59.46 (52.97–76.42)	51.19 (39.92–102.30)	0.949	53.98 (37.75–80.06)	71.99 (51.02–133.29)	0.442
**PON1 (IU/mL)**	10.24 (8.24–11.21)	10.36 (9.70–17.65)	0.322	10.93 (9.93–12.58)	18.27 (10.29–20.13)	0.037 *	11.90 (11.08–12.34)	11.94 (10.90–13.95)	0.542	11.35 (10.99–12.78)	11.11 (9.26–12.49)	0.460
**PROT (g/dL)**	5.38 (4.98–5.54)	5.38 (5.00–5.72)	0.585	6.16 (6.12–6.29)	6.00 (5.70–6.25)	0.203	5.97 (5.59–6.18)	6.04 (5.74–6.51)	0.514	5.56 (5.06–5.70)	6.03 (5.77–6.36)	0.104
**ALBU (g/dL)**	3.64 (3.58–3.70)	3.78 (3.61–3.92)	0.072	3.83 (3.54–4.03)	3.96 (3.77–4.08)	0.217	3.87 (3.74–4.08)	3.88 (3.72–4.06)	1.000	3.74 (3.73–3.83)	3.78 (3.62–3.89)	1.000
**GLOB (g/dL)**	1.66 (1.40–1.84)	1.58 (1.49–1.80)	0.824	2.32 (2.09–2.63)	2.02 (1.83–2.32)	0.130	2.00 (1.86–2.20)	2.17 (1.85–2.46)	0.542	1.73 (1.45–1.96)	2.24 (1.92–2.63)	0.130
**Tiol (µmol/L)**	256.40 (243.40–302.00)	245.65 (229.15–269.12)	0.233	232.15 (209.00–283.80)	275.10 (229.98–309.23)	0.312	316.70 (290.38–335.08)	283.67 (264.52–303.85)	0.145	256.20 (247.20–301.10)	277.50 (226.90–296.45)	0.879
**FRAP (mmol/L)**	0.35 (0.34–0.35)	0.29 (0.27–0.31)	<0.001 ***	0.30 (0.26–0.31)	0.34 (0.33–0.35)	0.001 ***	0.42 (0.39–0.43)	0.35 (0.31–0.37)	0.003 **	0.36 (0.32–0.37)	0.26 (0.25–0.28)	0.001 ***
**TEAC (mmol/L)**	0.74 (0.73–0.74)	0.67 (0.65–0.70)	<0.001 ***	0.73 (0.70–0.74)	0.76 (0.72–0.78)	0.072	0.80 (0.79–0.81)	0.78 (0.72–0.81)	0.480	0.76 (0.74–0.77)	0.73 (0.69–0.75)	0.234
**AOPP (µmol/L)**	41.00 (38.90–45.80)	56.15 (47.78–68.53)	<0.001 ***	47.70 (45.00–49.70)	48.85 (43.95–55.65)	0.686	57.00 (48.02–64.53)	48.45 (42.18–59.38)	0.184	48.40 (38.00–49.50)	53.70 (45.60–62.52)	0.104
**C2C (ng/mL)**	98.77 (43.36–147.52)	174.57 (75.47–262.50)	0.052	158.87 (150.14–187.70)	81.73 (59.08–140.31)	0.018 *	72.15 (59.16–98.95)	71.77 (47.41–110.60)	1.000	34.10 (27.64–72.07)	43.52 (39.00–68.28)	0.442
**HA (ng/mL)**	572 (329.70–572.19)	5255.08 (4131.26–6951.63)	<0.001 ***	1244.45 (708.85–1413.25)	9319.78 (7819.95–11142.59)	<0.001 ***	308.85 (33.75–583.46)	8388.45 (6289.35–11921.65)	<0.001 ***	39.85 (9.05–316.45)	39.85 (9.05–316.45)	0.006 **

* *p* < 0.05; ** *p* < 0.01; *** *p* < 0.001 (Wilcoxon rank-sum test). Biomarkers are shown in bold. CT = Control; NB = Nanobots + PRGF. T0 = before infiltration, T1 = 28 days post infiltration, T2 = 56 days post infiltration and T3 = 84 days post infiltration.

## Data Availability

The datasets used and/or analyzed for this study are available from the corresponding author upon reasonable request.

## Abbreviations

The following abbreviations are used in this manuscript:

ALBU	Albumin
AOPP	Advanced oxidation protein products
BIPED	Burden of disease, Investigative, Prognostic, Efficacy, Diagnostic
BMAC	Bone marrow aspirate concentrate
C2C	Type II collagen cleavage neoepitope
CRP	C-Reactive protein
CT	Control group
ELISA	Enzyme-linked immunosorbent assay
ERC	European research council
FDA	Food and drug administration
FRAP	Ferric reducing ability of plasma
GLOB	Globulins
HA	Hyaluronic acid
HDL	High-density lipoprotein
Hp	Haptoglobin
HyaNM	Hyaluronidase nanomotor
IA	Intraarticular
IBEC	Institute for bioengineering of catalonia
IGF-1	Insulin-like growth factor 1
IL-6	Interleukin-6
IM	Intramuscular
IO	Intraosseous
IQR	Interquartile range
IU	International units
MAPK	Mitogen-activated protein kinase
mCRP	Monomeric C-reactive protein
MMP	Matrix metalloproteinase
MSC	Mesenchymal stem cell
MSNP	Mesoporous silica nanoparticle
NB	Nanobots group
NF-κB	Nuclear factor kappa B
NM	Nanomotor
NSAID	Non-steroidal anti-inflammatory drug
OA	Osteoarthritis
PBS	Phosphate-buffered saline
pCRP	Pentameric C-reactive protein
PDGF	Platelet-derived growth factor
PON1	Paraoxonase-1
PRGF	Plasma rich in growth factors
PROT	Total protein
PRP	Platelet-rich plasma
ROS	Reactive oxygen species
SF	Synovial fluid
SPSS	Statistical package for social sciences
SYSADOA	Symptomatic slow-acting drugs for osteoarthritis
TEAC	Trolox equivalent antioxidant capacity
TGF-β	Transforming growth factor beta
Thiol	Total thiols
UrNM	Urease nanomotor
VEGF	Vascular endothelial growth factor
